# Left-Sided Acute Appendicitis in an Adult With Intestinal Malrotation: A Case Report From Jordan

**DOI:** 10.7759/cureus.97515

**Published:** 2025-11-22

**Authors:** Ramadan Hassanat, Mohammad Alhroot, Ahmad Alzboon, Ban Sha’ban, Shatha Dmour, Khaled Helael, Ahmad Telfah, Mohannad K Bawaneh, Bilal Rafiq, Saad Awraikat

**Affiliations:** 1 Department of General Surgery, Royal Medical Services, Amman, JOR; 2 Department of Radiology, Royal Medical Services, Amman, JOR; 3 Department of Anesthesiology, Royal Medical Services, Amman, JOR

**Keywords:** adult malrotation, intestinal malrotation, laparoscopic appendectomy, left-sided appendicitis, sma/smv inversion

## Abstract

Intestinal malrotation persisting into adulthood is rare and may cause atypical presentations of common surgical emergencies such as appendicitis. Left-sided acute appendicitis due to malrotation is an uncommon but important diagnostic pitfall. We present A 28-year-old woman with no past medical history who presented to the emergency department at our institution in August 2025 with a three-day history of progressive left-sided abdominal pain localized mainly to the left iliac fossa. Laboratory investigations revealed leukocytosis (WBC 15 × 10^9^/L) with neutrophilia. Contrast-enhanced CT demonstrated intestinal malrotation with a reversed superior mesenteric artery/superior mesenteric vein relationship, large bowel predominantly on the left side, and a left-sided, inflamed appendix with minimal surrounding fat stranding, minimal pelvic free fluid, and small ovarian cysts. The solid abdominal organs were unremarkable. Laparoscopic exploration revealed an inflamed appendix in the left iliac fossa with a healthy cecum and bowel base. Laparoscopic appendectomy was performed without performing the Ladd's procedure. The postoperative course was uneventful, and histopathology confirmed acute appendicitis with focal serositis. Intestinal malrotation in adults can shift the appendix to the left hemipelvis, leading to left-sided abdominal pain and diagnostic delay. Recognition of imaging features is crucial for diagnosis and surgical planning. Laparoscopic appendectomy provides safe and effective management.

## Introduction

Acute appendicitis is an emergency surgical condition presented with acute abdominal pain. The pathology is diagnosed clinically and rarely needs radiological assessment; the presenting features are variable according to the site of the appendix [1]. The patient presents with urinary symptoms if the appendix is pelvic, and features mimic cholecystitis if it's sub-hepatic, although in most cases the findings are located in the right iliac, but appendicitis can also be seen in unusual sites.

Intestinal malrotation is a congenital anomaly resulting from abnormal rotation and fixation of the midgut during embryogenesis. While commonly diagnosed in neonates, its persistence into adulthood is rare, occurring in approximately 0.2-0.5% of the population, and is often asymptomatic or discovered incidentally [1,2]. When complications arise-such as volvulus or appendicitis-diagnosis can be challenging.

Normally, the gut rotates 270 degrees counterclockwise around the superior mesenteric artery during gestation. There are three types of intestinal malrotation: nonrotation, where the small bowel in the right side and the colon in the left side. Partial rotation, where the duodenojejunal junction fails to cross the midline, and very rarely reverse rotation, where the colon is located posterior to the superior mesenteric artery [3]. Left-sided acute appendicitis (LSAA) due to malrotation is a particularly rare presentation, with an estimated incidence of 0.04% among appendicitis cases [3]. Because left lower quadrant pain often leads clinicians to consider diverticulitis, gynecologic, or urinary tract pathologies, LSAA can cause diagnostic confusion and delay. Cross-sectional imaging, particularly CT, plays an essential role in identifying malrotation and guiding surgical management [4,5]. Patients with malrotation are usually asymptomatic unless complicated by intestinal obstruction, diverticulitis, or appendicitis. Performing a laparoscopic appendectomy in most simple cases of an inflamed appendix is a sufficient procedure. In contrast, if the case was associated with intestinal obstruction or volvulus, further intervention, such as a Ladd's procedure and derotation of the bowel, may be necessary [3].

In the literature review, there are many case reports describing left-sided appendicitis, as in the case of situs inversus, or with a long tip appendix reaching the left iliac fossa, but a few cases described left-sided appendicitis in the case of malrotation, and the last one was in 2024 [6,7].

## Case presentation

A 28-year-old woman, with no significant past medical or previous abdominal surgeries, presented to the surgical emergency department at our institution in October 2025 with a three-day history of lower abdominal pain. The pain was described as dull, constant, and localized predominantly to the left iliac fossa. She also reported mild nausea and loss of appetite, but denied vomiting, urinary symptoms, or alterations in bowel habits. On examination, she was afebrile and hemodynamically stable. Abdominal palpation revealed tenderness and mild guarding in the left iliac fossa without rebound or rigidity. No masses were palpable, and pelvic examination was unremarkable. Laboratory studies showed WBC 15 × 10^9^/L with neutrophilia; electrolytes, liver, and renal function tests, and urinalysis were normal (Table 1).

**Table 1:** Complete blood count (CBC) results

Parameter	Result	Reference range	Interpretation
WBC	15 × 10^9^/L	4.0–10.0 × 10^9^/L	Elevated
Neutrophils	85%	40–75%	High (neutrophilia)
Lymphocytes	10%	20–45%	Low
Hemoglobin	13.2 g/dL	12.0–15.5 g/dL	Normal
Hematocrit	39%	36–46%	Normal
Platelets	280 × 10^9^/L	150–400 × 10^9^/L	Normal

**Figure 1:**
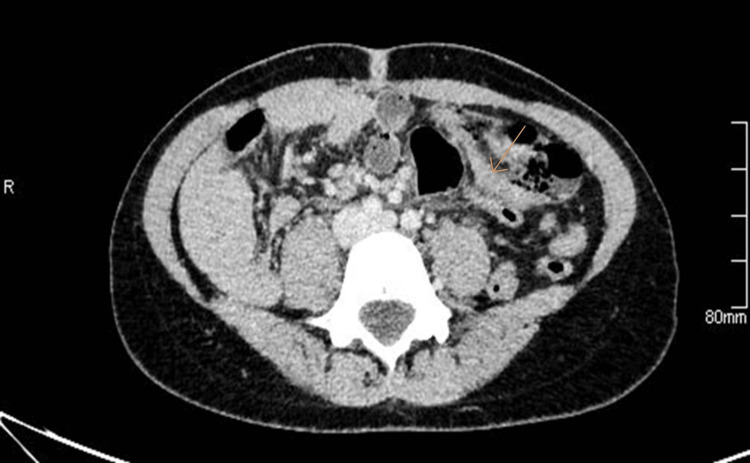
Axial CECT demonstrates a left-sided appendix arising from the malrotated cecum, measuring approximately 9 mm, with periappendiceal fat stranding, consistent with acute appendicitis. The arrow indicates the appendix arising from the malrotated cecum. CEDCT: contrast-enhanced computed tomography.

Gynecological examination and pelvic imaging were unremarkable. Transabdominal ultrasound demonstrated a normal-sized uterus with preserved myometrial echotexture, no adnexal masses or cystic lesions, and no evidence of free fluid in the pelvis. Contrast-enhanced computed tomography (CECT) of the abdomen and pelvis revealed intestinal malrotation, with the large bowel predominantly situated on the left side and a reversal of the superior mesenteric artery/superior mesenteric vein (SMA/SMV) relationship [6]. The appendix was visualized in the left lower abdomen, measuring approximately 9 mm in diameter, and demonstrated minimal periappendiceal fat stranding, consistent with early inflammation. There was trace pelvic free fluid and small ovarian cysts, while the liver, spleen, pancreas, adrenal glands, and both kidneys appeared unremarkable (Figure 1).

The findings were consistent with acute appendicitis in the setting of intestinal malrotation.

**Figure 2:**
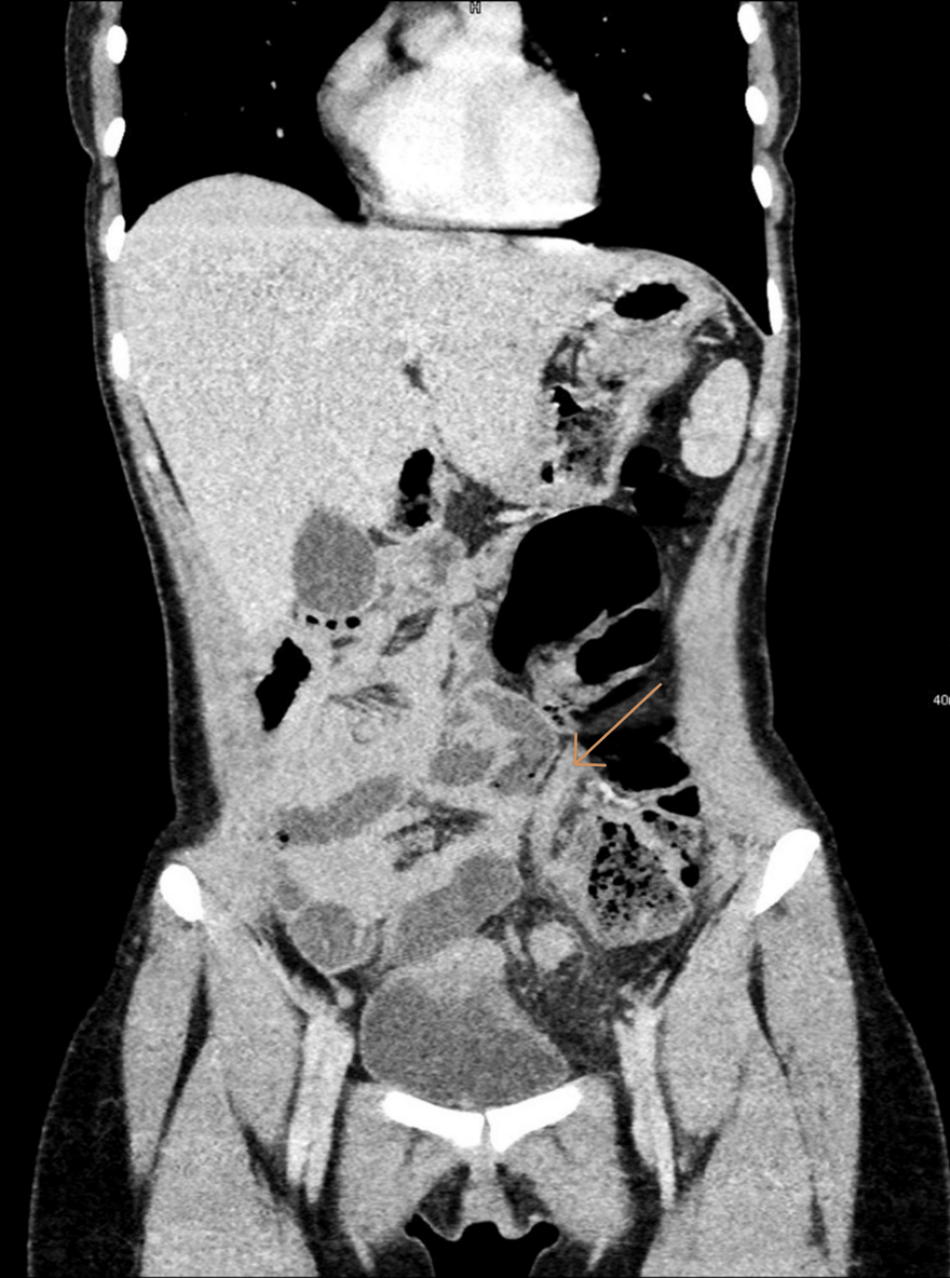
Coronal CECT demonstrating the appendix in the left lower quadrant (arrow), with the cecum and terminal ileum located abnormally on the left side. The SMV is seen left of the SMA, consistent with intestinal malrotation. Localized inflammatory changes are present without perforation or abscess formation. The arrow indicates the distal appendiceal tip. CECT: Contrast-enhanced computed tomography; SMA: superior mesenteric artery; SMV: superior mesenteric vein.

**Figure 3:**
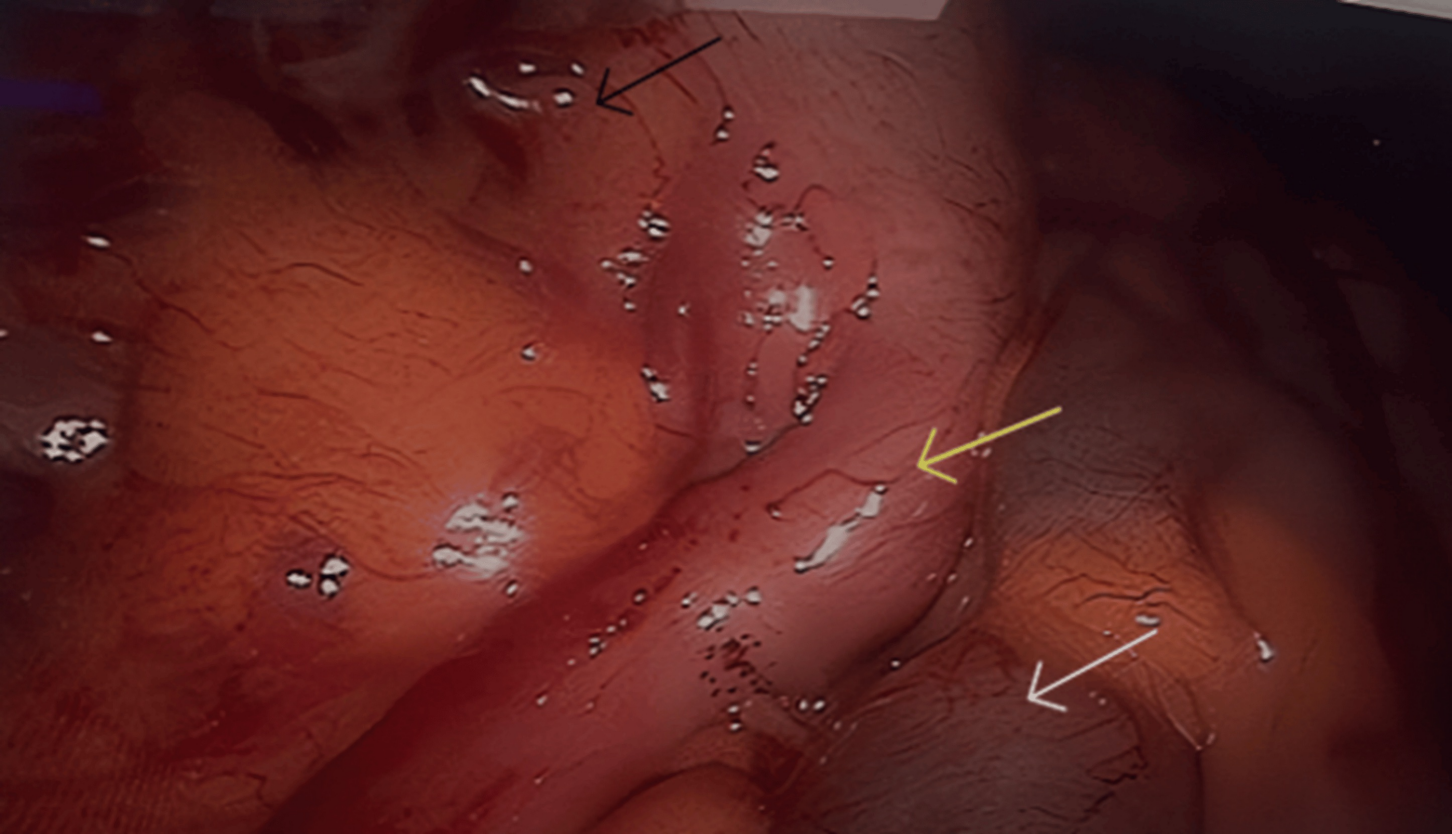
Laparoscopic view demonstrating left-sided abdominal anatomy in intestinal malrotation. The inflamed appendix is seen in the left iliac fossa (yellow arrow), with the cecum positioned on the left side of the abdomen (black arrow) and the small bowel predominantly on the right (white arrow).

**Figure 4:**
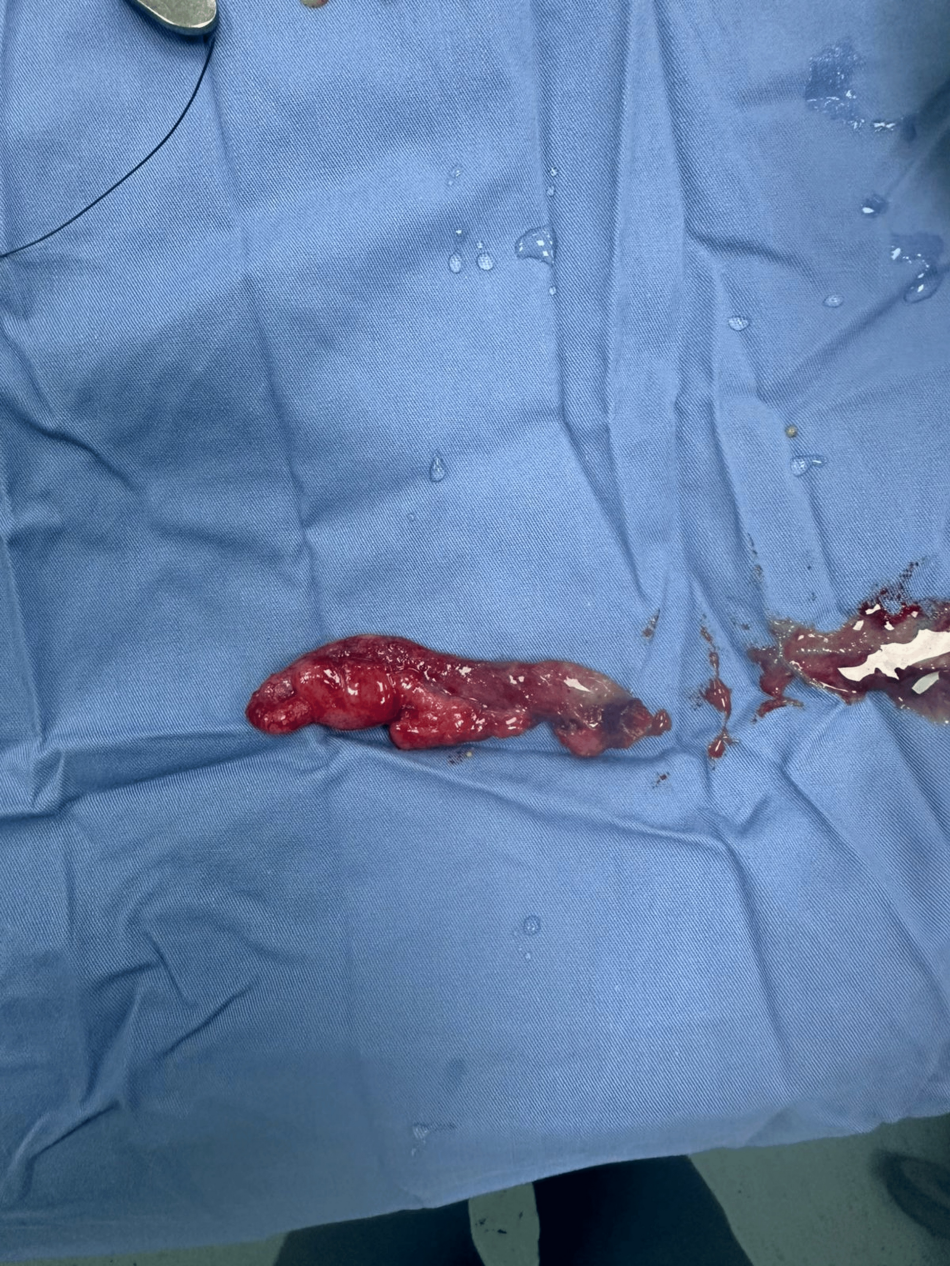
Gross appearance of the inflamed appendix following laparoscopic removal. The specimen shows an edematous, hyperemic appendix consistent with acute appendicitis in the setting of intestinal malrotation.

The patient underwent a laparoscopic appendectomy through three small incisions. A supraumbilical incision was used to create pneumoperitoneum and insert a 12-mm camera port, followed by a 12-mm working port in the left lower quadrant and a 10-mm port in the mid-lower abdomen for grasping and dissection. Intraoperatively, the appendix was inflamed and located in the left iliac fossa (yellow arrow), with the cecum on the left side (black arrow) and the small bowel predominantly on the right (white arrow), confirming intestinal malrotation. These findings were consistent with preoperative imaging demonstrating the SMV positioned to the left of the SMA. The bowel was viable, with no volvulus or obstructive Ladd’s bands. The appendix was dissected and removed without the need for additional procedures. The procedure was uneventful (Figures 2-4).

The postoperative course was smooth. The patient resumed oral intake the same day and was discharged on postoperative day two. Histopathology revealed acute appendicitis with focal serositis. At the two-week follow-up, she was asymptomatic and had fully recovered. Two weeks is a standard protocol in our institution for all appendectomy patients as a follow-up outpatient clinic, unless complications arise.

## Discussion

Left-sided acute appendicitis (LSAA) is a rare clinical entity that poses significant diagnostic challenges for clinicians and radiologists alike [2]. The classical presentation of appendicitis involves right lower quadrant pain, reflecting the normal anatomical position of the appendix in the right iliac fossa. However, in rare congenital anomalies such as intestinal malrotation or situs inversus totalis, the appendix may lie in an atypical position, leading to misleading clinical features and diagnostic delay [1,3].

Congenital intestinal malrotation results from abnormal midgut rotation during embryonic development, typically between the fifth and twelfth weeks of gestation [1]. Normally, the midgut undergoes a 270° counterclockwise rotation around the SMA. Failure or incomplete rotation can result in abnormal positioning of the small and large intestines, with the cecum and appendix often located on the left side [2]. Although malrotation is typically detected in infancy, it may remain asymptomatic until adulthood and be discovered incidentally during imaging or surgery. The estimated prevalence of malrotation in adults ranges from 0.0001% to 0.19%, and among these, LSAA represents less than 0.04% of all appendicitis cases [3,5].

Because of the abnormal anatomical location, LSAA often presents atypically. Patients commonly report left lower quadrant pain, which can be confused with conditions such as sigmoid diverticulitis, ovarian torsion or cyst rupture, ureteric colic, or pelvic inflammatory disease in females. In our patient, the absence of gastrointestinal or urinary symptoms and the localized tenderness in the left iliac fossa initially raised suspicion for a gynecological cause-demonstrating how atypical localization can obscure the diagnosis and delay appropriate management [3,8,9].

Radiological evaluation is critical in establishing the diagnosis. While ultrasonography is often used as a first-line imaging modality, it has limited sensitivity due to operator dependency and atypical organ positioning [10]. CECT remains the gold standard for diagnosis, providing detailed visualization of the intestinal orientation and appendiceal inflammation [4]. Key CT findings include abnormal bowel distribution (small bowel on the right and colon on the left), reversed SMA/SMV relationship, and an inflamed appendix with periappendiceal fat stranding and localized fluid [1,2,4,5]. Recent studies also highlight the 'absent retromesenteric duodenum' sign as a more reliable diagnostic indicator of malrotation, complementing vascular orientation in improving accuracy [2,4].

A hallmark of intestinal malrotation is the presence of Ladd’s bands-fibrous peritoneal attachments extending from the cecum or right colon across the duodenum to the retroperitoneum. These bands may cause extrinsic compression of the duodenum, leading to partial or complete intestinal obstruction [2,10]. The Ladd procedure, described in 1936, involves division of these bands, widening of the mesenteric root to prevent volvulus, repositioning of bowel loops [small bowel on the right, colon on the left], and usually appendectomy to prevent future diagnostic confusion [2,9,10]. In adults, prophylactic Ladd’s procedures for incidentally detected malrotation remain controversial. Current literature supports a selective approach: performing Ladd’s procedure when patients are symptomatic or when obstructive Ladd’s bands are found intraoperatively, and limiting management to appendectomy when malrotation is incidental and asymptomatic [9,10].

Several recent case reports have highlighted the diagnostic and therapeutic nuances of LSAA. Feeney et al. reported a laparoscopically managed case of left-sided appendicitis due to malrotation confirmed by CT [5]. Aassouani et al., Hoang et al., and Al Sharqi et al. described similar presentations across age groups and anatomical variants [11-13]. Assefa et al. reported a perforated LSAA following delayed diagnosis, while Silva et al. documented uncomplicated laparoscopic management [1,2,4-6,8,14]. Since Akbulut’s comprehensive review, approximately 30-40 new LSAA cases have been reported worldwide [15]. Most were diagnosed by CT and managed laparoscopically, demonstrating the effectiveness of cross-sectional imaging and minimally invasive techniques in early diagnosis and treatment.

Laparoscopic appendectomy remains the preferred approach for LSAA [16]. It provides diagnostic clarity, allows exploration of the entire abdominal cavity, and enables treatment in a single procedure. If obstructive Ladd’s bands or volvulus are identified intraoperatively, conversion to a Ladd procedure should be considered [10]. The prognosis is excellent with early diagnosis and intervention, while delayed recognition can lead to perforation, abscess, or sepsis. Mahajan et al. reported that diagnostic delays exceeding 48 hours doubled the risk of perforation compared to typical right-sided appendicitis [9,17]. Our patient’s clinical presentation, imaging findings, and operative outcome align closely with previously reported cases, adding to the literature documenting successful laparoscopic management of LSAA in adult females [1,5,6].

## Conclusions

Left-sided abdominal pain should not exclude appendicitis. In adults, intestinal malrotation can cause left-sided appendicitis, posing a diagnostic challenge. Awareness of CT features-particularly superior mesenteric artery/superior mesenteric vein inversion and an abnormal duodenal course-is key to prompt diagnosis. Laparoscopic appendectomy offers a safe and effective treatment, and the decision for a Ladd procedure should be individualized based on symptoms and intraoperative findings.
